# Health insurance coverage and access to child and maternal health services in West Africa: a study protocol for a systematic review

**DOI:** 10.1186/s13643-021-01628-2

**Published:** 2021-03-11

**Authors:** Joshua Dadjo, Olumuyiwa Omonaiye, Sanni Yaya

**Affiliations:** 1 Interdisciplinary School of Health Sciences, University of Ottawa, Ottawa, Ontario Canada; 2 Centre for Quality and Patient Safety Research, School of Nursing and Midwifery, Deakin University, Burwood, Melbourne, Victoria Australia; 3 Centre for Nursing and Midwifery Research, James Cook University, Townsville, Queensland Australia; 4 School of International Development and Global Studies, University of Ottawa, Ottawa, Ontario Canada; 5 The George Institute for Global Health, Imperial College London, London, UK

**Keywords:** Maternal and child, Insurance, Health coverage, West Africa, Services

## Abstract

**Background:**

Though many studies have discussed the impact of health insurance on access to medical services, few have considered Western Africa. Despite decades of targeted efforts, West Africa has the most elevated maternal mortality rates (MMR) and under-five mortality rates in the world. The solution to this issue is widely believed to be the implementation of universal health coverage (UHC) as most causes of death could be effectively dealt with through primary health care providers. It is possible that UHC without additional efforts to tackle important determinants of health such as education and poverty is insufficient. The objective of this study is to examine the link between being covered by health insurance and access to health services for mothers and children in West Africa.

**Methods:**

A systematic literature review will be conducted. We will search the online databases MEDLINE complete, Embase, CINAHL complete, and Global Health from inception onwards. The focus will be on primary research studies and grey literature that examined health insurance in relation to access to maternal and child health services. Two reviewers will independently screen all citations, full-text articles, and abstract data. The primary outcome will be maternal and child access to health insurance and access to primary and secondary services such as attending the minimum number of prenatal visits and accessing services in emergency circumstances where catastrophic expenditures may have been an obstacle. A standardized data extraction form by the Cochrane library will be used. A narrative synthesis will be conducted with a summary of findings tables to be produced.

**Discussion:**

The systematic review will present findings on the impact of access to health insurance on access to maternal and child health care. The findings will inform discussion around the pursuit of UHC as a key health systems policy. The final manuscript will be disseminated through peer-reviewed journal and scientific conferences.

**Systematic review registration:**

PROSPERO CRD42020203859

**Supplementary Information:**

The online version contains supplementary material available at 10.1186/s13643-021-01628-2.

## Background

In September of 2000, the 191 member states of the United Nations signed the Millennial Development Goals [1]. Included in these goals was the reduction of child mortality and improving maternal health by 2015. A decade and a half of efforts and investments that followed led to the decrease of maternal mortality rates (MMR) ratio and under-five deaths in children by more than half. Additionally, the number of women who benefited from skilled birth assistance rose by 59% between 1990 and 2014 [1]. Despite these efforts, by 2015, only about half of the women globally received the recommended amount of antenatal care [1]. Then came the 17 Sustainable Development Goals (SDG), signed by the United Nations in 2015 with the intention to continue the expansion of the work done in the previous 15 years. The third goal, *Ensure Healthy Lives and Promote Well-Being at All Ages* includes targets such as the continued reduction of the under-five mortality and MMR rates, as well as expansion of access to contraceptives, lowering the adolescent fertility rate and expanding access to immunization [2]. So far, billions of dollars have been pledged or invested towards these goals, including a $1.4 billion commitment over 10 years by the Canadian government [3]. Specifically, the SDG set out to achieve the following targets in relation to maternal and child health: the reduction of under-five mortality to less than 25 in 1000, neonatal mortality lowered to less than 12 in 1000, and MMR to 70 per 100 000 live births as targets [2].

Western Africa is known to have the world’s highest MMR rates in the world [4] and one of the highest rates for under-five mortality in children, including at the neonatal stage [5]. The MMR in Western Africa range from the low 300 in 100 000 live births in Benin and Ghana to around 500 in Cameroon and Niger to over 900 per 100 000 live births in Nigeria [6]. The unfortunate reality is that most deaths are preventable. When it comes to mothers, hemorrhage, exacerbation of pre-existing conditions by pregnancy, eclampsia, and sepsis are major causes of maternal deaths [7]. Whereas for children, specifically newborns, the main cause of death are complications during birth, such as intrapartum events, preterm births, or infections such as diarrhea and sepsis [8]. In both cases, appropriate and timely medical care is the solution through mostly low-tech and cost-effective technologies [8] that are often accessible when a mother is giving birth in the presence of trained and skilled health workers in government-approved health facilities.

UHC is defined as the ability for people to access and use all necessary health services at a substantial quality without incurring catastrophic financial costs [9]. Specifically, the World Health Organization (WHO) sets out equity in access, strong quality of health services, and protection against financial risk as important tenants of UHC [10]. Therefore, the goals of UHC include increased access to essential medical services, while decreasing the rate of catastrophic costs to consumers. A strong argument for UHC is its role in Malawi [11]. Malawi ranked 174 out of 187 on the Human Development Index, with a GDP of US$ 494.40 per capita [11]. The implementation of UHC was marked by policies targeting areas with poor indicators through increased funding, training, and retraining health care staff and human resources [12]. These efforts have led to an increase in overall coverage and stronger health indicators, such as lowering the under-five mortality rate [12].

The impact of health insurance coverage on access to maternal services has been considered in some regions of West African countries, though there are only a handful of studies available. As in most low- and middle-income countries, many West African countries have used community-based health insurance schemes or social schemes [13]. Two regions that have been studied are the Wa East and West regions of Northern Ghana [14] and Kawara State in Nigeria [15]. Further, one relevant study has been carried out in Mauritania [12]. In 2003, Ghana introduced a National Health Insurance Service [15]. To increase access to the services, the Ghanaian government exempted certain groups, including pregnant women, from paying premiums. The study focused on the Wa East and Wa West districts of Northern Ghana, two of the poorer regions of the country. In this study, over 90% of women were enrolled in the National Health Insurance Service. Of those enrolled, 98.6% utilized antenatal services, and the majority, 66.5%, gave birth in health facilities. Notably, these increases were shared among various social and economic classes. Furthermore, in Nigeria, an interrupted time-series study was conducted to determine the impact of the state health insurance scheme, one that was developed through a public-private partnership [15]. Findings from this study showed that when women had access to services and insurance, rates of insurance increased significantly, from 0 to 70.2% and in hospital deliveries increased by 62%. This study noted that it is not clear if health insurance coverage without the elimination of other barriers, such as distance to services, will be sufficient to guarantee access and utilization of health services. In 2002, Maurtiania developed an obstetrical risk insurance (ORI) [12]. Unique to other models, this scheme did not depend on risk pooling, but rather on pre-payment. Women could pay 16–18 USD to guarantee access to a core set of services. The study observed that only 58% of women interviewed knew ORI existed and of those who knew, two thirds enrolled. The study observed that for those insured, there was a moderate increase in pre-natal use of services. Yet, ORI had no meaningful impact on cesarean section rates, postnatal care, and neonatal mortality. This study would suggest that pre-payed schemes, a type of community-based scheme [13], may have their limitations, indicating that various schemes would have various implications on the rates of utilization of services. Further, efforts around recruitment into schemes, especially regional schemes, would impact the rates of adhesions to schemes, in turn impacting the rates of access and utilization of services.

Despite decades of targeted public health interventions, the West African region still has the most elevated MMR and under-five mortality rates in the world. The solution to this issue is widely believed to be the implementation of UHC [16, 17], as most causes of death could be effectively dealt with through primary care providers. Yet, little is known about the impact of current efforts in increasing access to services based on UHC programs. Hence, this study’s primary objective will be to conduct a systematic review of published studies that have examined the link between health insurance coverage and access and utilization of health care services for mothers and newborns in Western Africa. Secondary objectives are to identify evidence gaps relating to barriers in coverage.

## Methods

### Study design

The study will be a systematic review of published studies that consider the relationship between access to health insurance and access to health services for mothers and children aged 0–5 years old in West African countries. This review was reported following the Preferred Reporting Items for Systematic Reviews and Meta-Analyses Protocols (PRISMA-P) criteria [18] (see Supplementary file 1). The protocol has been registered within the International Prospective Register of Systematic Reviews (PROSPERO) database (registration number CRD42020203859).

### Research questions

This review will address the following questions: What is the impact of catastrophic expenditure on access and utilization of health care services for mothers and newborns in Western Africa? What is the impact of health insurance on access and utilization of services for mothers and newborns in Western Africa?

### Eligibility criteria

Studies will be selected according to the following criteria: population, interventions and comparators, outcomes, and study design.

### Population

The review will consider studies that focus on mothers and children under the age of 5 years old in West African countries (see list of countries in Supplementary file 2). It is likely that the majority of studies available will focus on the experience of mothers who have recently given birth. There will be an inclusion of women and children from every economic and social class, and given that the population of women in West Africa is overwhelmingly poor and rural, the study will likely have an outsized consideration of this sub-population.

### Interventions and comparators

The intervention considered will be whether or not mothers have access to health services once they are insured. Health insurance coverage is accessible through various forms in West African countries. These include national schemes funded by taxpayers and managed by public authorities and community-based health insurance schemes funded by community members and administered by religious, not-for-profit, or other smaller scale-organization [13]. The funding of insurance schemes will not be a factor that leads towards exclusion. All health services will be considered, though special attention will be paid to prenatal and neonatal services.

### Outcomes of interest

The primary outcome of interest will be findings in the literature on the impact of health insurance on access to primary and secondary maternal and child health services. For example, relevant findings include whether a mother with health insurance attended antenatal services or whether a child received immunization. This would also take into consideration whether catastrophic expenditure remains a barrier. Secondary outcomes would include explicit gaps in the literature where a study may examine this question but not make any conclusion. There would also be interest in factors that may impact the primary findings, such as distance to services, education wealth, or other social determinants of health.

### Study design

The review will include observational studies such as cohort studies, cross-sectional surveys, and longitudinal studies that consider the impact of health insurance status on access to health services. Studies with controlled interventions will be excluded unless they are observational only studies conducted published in peer-reviewed journals in English or in French will be included in the review.

### Information sources and search strategy

With the assistance of an experienced librarian with expertise in health and social sciences, we will search the electronic databases, MEDLINE complete, Embase, CINAHL complete, and Global Health. A range of terms and combinations will be used with MeSH terms and/or text words. Studies will be identified using a combination of subject headings and keywords unique to each database. Concepts pertaining to access to health services, maternal health, child and newborn health, and health insurance coverage in West Africa will be searched. In order to broaden the results, terms relevant to each period in the continuum of care will be included. Reference lists of included studies will be perused to identify any additional studies that may satisfy the eligibility criteria. A draft of the search strategy for MEDLINE is provided in Supplementary file 3.

### Screening and selection process

All results will be sent to Covidence (Veritas Health Innovation Inc.), and duplicates will be removed automatically. The titles and abstracts will be screened independently by one of the reviewers (JD). Full-text review of articles retained after the title and abstract screening will be undertaken independently by two reviewers (JD and OO), and conflicts between JD and OO will be resolved by SY. Following the full-text screening, the list of included studies will be reviewed, screened by JD, OO, and SY and retrieved if eligible for the review, with the process continuing until no new articles are identified. Finally, articles that have cited the selected text will be obtained using the database SCOPUS to identify other relevant articles that may have been missed.

### Assessment of methodological quality

The methodological quality in the studies that will be selected for retrieval will be independently evaluated by two authors following full-text screening. The assessments will be done using Joanna Briggs Institute (JBI) critical appraisal tools for observational studies [19]. The quality of the studies will be reported in the narrative review as low, moderate, or high quality. Any disagreements between the two reviewers will be resolved through discussion and with the assistance of a third reviewer.

### Data extraction

The authors will adapt and develop data collection forms based on the needs of the review from a standardized data extraction form [20]. The forms will ensure data extraction is as consistent as possible across all studies, as the extracted data is used to synthesize the findings. We will use the forms to extract the following information from each article: (i) study setting (country), (ii) study aim(s), (iii) sample characteristics, (iv) data collection methods, (v) rates of coverage, (vi) rates of utilization and access to services for mothers and newborns, (vi) other factors and barriers that affect rates of access and utilization, (vii) types of coverage, and (viii) cost of coverage to the consumer.

### Data synthesis

The narrative synthesis without meta-analysis is chosen as the method of synthesis following considerations of time, resources, and appropriateness for addressing the aims of this review. It is useful in describing the differences between findings and identifying commonalities within and across groups in a large number of studies [21]. The thematic analysis provides the best way to organize and summarize findings in a concise manner from the large body of evidence [21]. This review will synthesize the obtained evidence on the impact of health insurance coverage on access to services for mothers and young children in West Africa. Summaries of a consistent format will be developed for every study utilized. The format consists of the context of the study, the main findings in relation to the review, and other extenuating factors to be considered. The summaries will be provided in a table where appropriate. For greater clarity, the “synthesis without meta-analysis (SWiM) in systematic reviews” will be used [22]. As mentioned, a meta-analysis will not be included due to the concern that we will obtain a small number of articles [23]. A secondary reason is though the comparator will be the same throughout the studies, the specific health services received will vary from study to study. This variation would provide great of a diversity in findings to allow for a proper meta-analysis [24].

### Confidence in cumulative evidence

The strength of the body of evidence will not be assessed.

## Discussion

Though many studies have discussed the impact of health insurance on access to medical services, few have considered Western Africa as a whole. Despite decades of investments and targeted work, this region continues to have the most elevated rates of MMR and under-five mortality in the world. Many countries or regions employ some variation of community-based and national insurance schemes [13], yet the evidence on their impact on access to services for mothers and children is scarce.

Based on previous literature that carried out similar investigations in other settings, it is expected that decreased exposition to catastrophic health expenditure is likely to increase access and utilization of health services. This review will be the first to link coverage status to the utilization of services in the whole of West Africa. This protocol outlines the methodology and rationale while presenting examples of the limited evidence available. The strength of this paper will be the broad search for evidence, from inception to August 2020, in many journals and platforms, in both French and English. Limitations include the underrepresentation or underreported data points due to the limited number of studies on this issue. This may result in publication bias and methodological quality issues. Therefore, we will assess meta-bias(es) to ensure rigidity in the findings.

This study is likely to face few practical or operational issues. Access to data is facilitated by the Demographic Health Statistics. The methodology uses software and databases easily accessible. The COVID-19 pandemic could impact the timeline of this study, though it is unlikely it will.

Any amendment to this protocol while conducting the study will be outlined in PROSPERO and in the final manuscript.

The completed review will be published in a journal in order to effectively communicate the results to a range of stakeholders such as policymakers including health economist on the value of continued efforts to provide UHC in West Africa and the presumed need to combine UHC with policies that improve other social and economic indicators. Any important changes to the protocol will be submitted to the journal in which it was published.

## Supplementary Information

**Additional file 1:.** PRISMA Checklist.

**Additional file 2:.** List of eligible countries.

**Additional file 3:.** Draft Search terms.

## Abbreviations

CHE: Catastrophic health expenditure
MDG: Millennium Development Goals
MMR: Maternal mortality ratio
IO: International organizations
SDG: Sustainable Development Goals
WHA: World Health Assembly
WHO: World Health Organization
