# Supplementary material for: Health insurance coverage and access to child and maternal health services in West Africa: a study protocol for a systematic review
Source: Syst Rev. 2021 Mar 11;10:74. doi: 10.1186/s13643-021-01628-2 (PMC7950425; doi:10.1186/s13643-021-01628-2)
Supplement: Supplementary file 2 — Additional file 2:. List of eligible countries. [file 13643_2021_1628_MOESM2_ESM.docx]

**Supplementary file 2**

**List of West African Countries**

| Benin |
| --- |
| Burkina Faso |
| Cape Verde |
| Gambia |
| Ghana |
| Guinea |
| Guinea-Bissau |
| Ivory Coast |
| Liberia |
| Mali |
| Mauritania |
| Nigeria |
| Niger |
| Saint Helena |
| Senegal |
