# Supplementary material for: Health insurance coverage and access to child and maternal health services in West Africa: a study protocol for a systematic review
Source: Syst Rev. 2021 Mar 11;10:74. doi: 10.1186/s13643-021-01628-2 (PMC7950425; doi:10.1186/s13643-021-01628-2)
Supplement: Supplementary file 3 — Additional file 3:. Draft Search terms. [file 13643_2021_1628_MOESM3_ESM.docx]

**Supplementary file 3**

Ovid **MEDLINE**(R) ALL 1946 to September 12, 2020 Strategy formed on September 11, 2020

| 1 | Mothers/ |
| --- | --- |
| 2 | (mother? or maternal).ti,ab. |
| 3 | exp Infant/ |
| 4 | Child, Preschool/ |
| 5 | (infant? or toddler? or newborn? or child*).ti,ab. |
| 6 | (preschool adj3 child*).ti,ab. |
| 7 | 1 or 2 or 3 or 4 or 5 or 6 |
| 8 | exp Insurance, Health/ |
| 9 | (health adj3 (insurance or coverage)).ti,ab. |
| 10 | insurance.ti,ab. |
| 11 | 8 or 9 or 10 |
| 12 | exp Africa, Western/ |
| 13 | west* africa.ti,ab. |
| 14 | 12 or 13 |
| 15 | exp Health Services/ |
| 16 | perinatal care/ or prenatal care/ |
| 17 | ((perinatal or prenatal) adj3 care).ti,ab. |
| 18 | (health adj2 (service? or care)).ti,ab. |
| 19 | (maternal adj2 health).ti,ab. |
| 20 | exp Maternal Health Services/ |
| 21 | 15 or 16 or 17 or 18 or 19 or 20 |
| 22 | 7 and 11 and 14 and 21 |
| 23 | exp Female/ |
| 24 | exp Pregnancy/ |
| 25 | exp Delivery, Obstetric/ or exp Developing Countries/ |
| 26 | exp Prenatal Care/ed, og, st, sn, td [Education, Organization & Administration, Standards, Statistics & Numerical Data, Trends] |
| 27 | exp Senegal/ |
| 28 | exp Benin/ |
| 29 | exp Burkina Faso/ |
| 30 | exp Ivory Coast/ |
| 31 | exp Gambia/ |
| 32 | exp Ghana/ |
| 33 | exp Liberia/ |
| 34 | exp Mali/ |
| 35 | exp Nigeria/ |
| 36 | exp Sierra Leone/ |
| 37 | exp Togo/ |
| 38 | exp Cape Verde/ |
| 39 | exp Guinea/ |
| 40 | exp Guinea-Bissau/ |
| 41 | exp Mauritania/ |
| 42 | exp Niger/ |
| 43 | exp Saint helena/ |
| 44 | Côte d’Ivoire |
| 45 | 7 or 23 or 24 |
| 46 | 14 or 27 or 28 or 29 or 30 or 31 or 32 or 33 or 34 or 35 or 36 or 37 or 38 or 39 or 40 or 41 or 42 or 43 |
| 47 | 21 or 25 or 26 |
| 48 | 11 and 45 and 46 and 47 |
